# The expression and clinical significance of melanoma-associated antigen-A1, -A3 and -A11 in glioma

**DOI:** 10.3892/ol.2013.1351

**Published:** 2013-05-15

**Authors:** LIRU GUO, MEIXIANG SANG, QINGRUI LIU, XIAOJIE FAN, XIAO ZHANG, BAOEN SHAN

**Affiliations:** 1 Department of Neurology, The Fourth Clinical Hospital of Hebei Medical University, Shijiazhuang, Hebei 050011, P.R. China; 2 Research Center, The Fourth Clinical Hospital of Hebei Medical University, Shijiazhuang, Hebei 050011, P.R. China; 3 Tumor Research Institute, The Fourth Clinical Hospital of Hebei Medical University, Shijiazhuang, Hebei 050011, P.R. China

**Keywords:** melanoma-associated antigen-A1, melanoma-associated antigen-A3, melanoma-associated antigen-A11, glioma, prognosis

## Abstract

Melanoma-associated antigens (MAGEs) were initially identified in melanoma and have since been widely studied. Melanoma-associated antigen-As (MAGE-As), a subfamily of MAGEs, are expressed in germ cells and various types of cancer, and are considered to be ideal targets for cancer immunotherapy. Glial cells and melanocytes originate from the neural ectoderm, so tumors derived from these two types of cells, i.e. gliomas and melanomas, may have common biological characteristics. However, studies on the expression of the MAGE-A family in gliomas are limited and conflicting. In the present study, the expression levels of MAGE-A1, -A3 and -A11 were detected by immunohistochemistry, and the association of their expression levels with the clinicopathological parameters, overall survival (OS) and ki-67 labeling indices of glioma patients were analyzed. The results showed that i) the expression levels of MAGE-A1, -A3 and -A11 proteins in the glioma tissues were 64.1, 51.3 and 57.7%, respectively and that no MAGE-A1, -A3 or -A11 expression was detected in the normal brain specimens; ii) the expression levels of MAGE-A1 and -A11 increased with ascending pathological grades and were positively correlated with the ki-67 labeling index; and iii) the OS of the patients in the groups with high MAGE-A1 (P=0.005) and -A11 (P=0.019) expression was statistically lower compared with the groups with low expression and no significant differences in OS were detected between the patients in the groups with high and low MAGE-A3 expression (P=0.304). Based on these results, we conclude that MAGE-A1, -A3 and -A11 may be used as ideal targets for glioma immunotherapy, and that MAGE-A1 and -A11 expression may be involved in tumor cell proliferation. These proteins may be potential indicators of a poor prognosis in glioma patients.

## Introduction

Gliomas are the most common primary tumors of the central nervous system in humans and are characterized by a rapid infiltrative growth pattern that makes complete surgical resection impossible. Despite progress in tumor diagnosis and treatment, including surgery, radiotherapy and chemotherapy, the median survival time is only one year and few patients survive for two years (1). Moreover, these conventional therapies often damage the surrounding normal brain tissues, leading to consciousness disorders and neurological deficits. Immunotherapy is a more effective and specific therapeutic method (2). Therefore, the identification of several biomarkers, which are expressed differentially in high-grade gliomas, low-grade gliomas and normal brain tissues, is urgently required for immunotherapy and the formation of a prognosis.

Cancer/testis antigens (CTAs) are a group of tumor-associated antigens that are expressed in normal testis germ cells, the placenta, trophoblasts and in various tumors (3–5). These antigens may be used as ideal targets for cancer immunotherapy due to their characteristic expression pattern (6). Melanoma-associated antigens (MAGE) are a subgroup of CTAs that include >60 genes in humans (7). The MAGE family is subdivided into two groups, MAGE-I and MAGE-II, based on their gene structure and tissue-specific gene expression (8). The MAGE-I group includes the MAGE-A, -B and -C subfamilies. The MAGE-A family is located in the q28 region of the X chromosome and includes 12 family members, named MAGE-A1 to A12 (9,10). Glial cells and melanocytes originate from the neural ectoderm, so tumors derived from these two types of cells, i.e. gliomas and melanomas, may have common biological characteristics. Although MAGEs have been well studied for >20 years in melanomas (11), these antigens have not been well characterized in gliomas. Kuramoto investigated the expression of the MAGE-A1 and -A4 proteins in 28 brain tumor tissues (14 gliomas and 14 non-gliomas) by immunoblot analysis and observed positive results in the majority of gliomas (12 of 14) and a few (5 of 14) non-gliomas (12). Bodey *et al* studied the expression of MAGE-A1 protein in childhood astrocytomas using an immunocytochemical method and observed that positively-stained cells were present in high-grade, but not low-grade, astrocytomas, suggesting that MAGE-A1 may be an indicator of childhood astrocytoma progression (13). Syed *et al* analyzed the composite expression of CTA and melanocyte-differentiation antigens (MDA) using RT-PCR in malignant gliomas and noted that the frequencies of MAGE-A3, -A1 and -A4 were 22, 16 and 7%, respectively (14).

In the present study, formalin-fixed and paraffin-embedded tissues and the clinicopathological parameters from 78 patients with glioma were collected and the expression levels of the MAGE-A1, -A3, -A11 and ki-67 proteins were evaluated by immunohistochemistry. Furthermore, the associations of patient prognosis and clinicopathological parameters with the expression of the MAGE-A1, -A3 and -A11 proteins were investigated. To the best of our knowledge, this is the first study to detect MAGE-A11 expression in gliomas and to show a correlation between its expression level and patient prognosis.

## Materials and methods

### Clinical specimens

A total of 78 glioma specimens obtained from patients who underwent surgical treatment at the Department of Neurosurgery of the Fourth Clinical Hospital of Hebei Medical University (Shijiazhuang, Hebei, China) between 2006 and 2010 were analyzed in the present study. No patients underwent any treatments, including radiotherapy or chemotherapy, prior to surgery. The patients included 45 males and 33 females with a mean age of 49.8 years (range, 22–79 years). Gliomas were classified according to the guidelines of the 2000 WHO classification (15). These tumors included nine grade I (pilocytic astrocytomas), 16 grade II (astrocytomas), 17 grade III (anaplastic astrocytomas) and 36 grade IV (glioblastoma) gliomas. Six normal human testis specimens were obtained as positive controls from patients with prostatic cancer who underwent surgical castration orchiectomy at the Department of Urinary Surgery, the Fourth Clinical Hospital of Hebei Medical University in 2007. A total of 15 normal brain specimens were obtained as negative controls from the donations of individuals who had succumbed due to injuries caused by traffic accidents. After surgery, the specimens were sent to the pathology department to be fixed with formalin and embedded in paraffin for conventional hematoxylin and eosin (HE) staining and immunohistochemical analysis. Informed consent was obtained from all recruited subjects prior to enrollment and all patients were consecutively enrolled. The study was approved by the Medical Ethics Committee of the Fourth Clinical Hospital of Hebei Medical University. All patients were followed up until September 2012.

### Clinicopathological parameters

The clinicopathological parameters, including age, gender, Karnofsky performance scale (KPS) score, histological types and pathological grades, were collected by reviewing medical records and telephone interview information.

### Immunohistochemistry and evaluation

Tissue sections of a 4-*μ*m thickness were cut from paraffin-embedded tissue blocks, mounted on silanized slides and incubated for 120 min in a thermostat at 60°C. The sections were then deparaffinized in xylene, rehydrated in sequential alcohol grades and washed with phosphate-buffered saline (PBS; pH 7.2) for 5 min three times. Antigen retrieval was performed by heating the sections in a microwave oven for 20 min in 10 mM sodium citrate buffer (pH 6.0) followed by endogenous peroxidase, using 3% hydrogen peroxide for 20 min. Subsequent to being washed in PBS, the samples were incubated in 10% normal goat serum at 37°C in a humidified chamber for 30 min to minimize non-specific protein binding. The sections were then incubated with rabbit-anti-human MAGE-A1 monoclonal antibody (1:200; Epitomics, Burlingame, CA, USA), rabbit-anti-human MAGE-A3 polyclonal antibody (1:100; Abcam, Cambridge, UK), rabbit anti-human MAGE-A11 polyclonal antibody (1:100; Epitomics) or mouse anti-human ki-67 monoclonal antibody (Jinqiao, Beijing, China) at 4°C overnight, followed by biotinylated secondary antibodies for 30 min at 37°C. A streptoavidin-biotinylated horseradish peroxidase-based detection system was used to detect antigen-specific binding. Normal rabbit or mouse IgG replaced the primary antibody for the controls. Finally, the slides were counterstained with HE for microscopic observation and evaluation.

To evaluate MAGE-A1, -A3 and -A11 expression in the various grades of glioma, qualitative and quantitative evaluations of the percentage of positive cells and the intensity of staining were performed using a light microscope at high magnification (×400) by examining 10 randomly selected visual fields per slide. The percentage of the antigen-positive cells were scored as follow: 0, no positive cells; 1, 0–10% positive cells; 2, 11–50% positive cells; and 3, >50% positive cells. The intensity of the staining was scored as follows: 0, no staining; 1, weak staining; 2, mild staining; and 3, high intensity staining. The final score per slide was the cross product of the scores of staining intensity and percentage of positive cells (16). The expression level was defined as between - and 3+, as follows: -, score <2; +, score of 2–3; 2+, score of 4; 3+, score of 6 or 9. A score of - or + was defined as a low expression level. A score of 2–3+ was defined as a high expression level.

The ki-67 labeling index (percentage of ki-67 positive cells) was examined by light microscopy at low magnification (×200) by observing 10 randomly selected visual fields. The average count of each field was the percentage of immunopositive neoplastic cells. Marked nuclear staining was regarded as positive and weak nuclear or cytoplasmic staining was negative. All samples were scored by two independent experienced pathologists. A high ki-67 labeling index was defined as when ≥10% of neoplastic cells were positive (17).

### Statistical analysis

Statistical analysis was performed using SPSS 11.5 software (SPSS Inc., Chicago, IL, USA). Two-sided tests were used to determine the significance and P<0.05 was considered to indicate a statistically significant difference for all statistical tests. The data are expressed as the mean ± SD of the experiments. Chi-squared or Fisher’s exact tests were used to evaluate the statistical significance of the differences and associations between MAGE-A1, -A3 and -A11 and the clinicopathological parameters. The correlations between MAGE-A1, -A3 and -A11 and the ki-67 labeling index were assessed by Spearman’s rank correlation coefficient. Overall survival (OS) time was defined as the period between the date of surgery to the date of mortality. The Kaplan-Meier method was used for the survival analyses. The statistical significance of the differences between the groups was evaluated using the log-rank test. The associations between OS and the potential prognostic factors were analyzed using Cox-regression multivariate analysis.

## Results

### Expression of MAGE-A1, -A3 and -A11 in glioma and normal brain tissues

**Figure 1.**
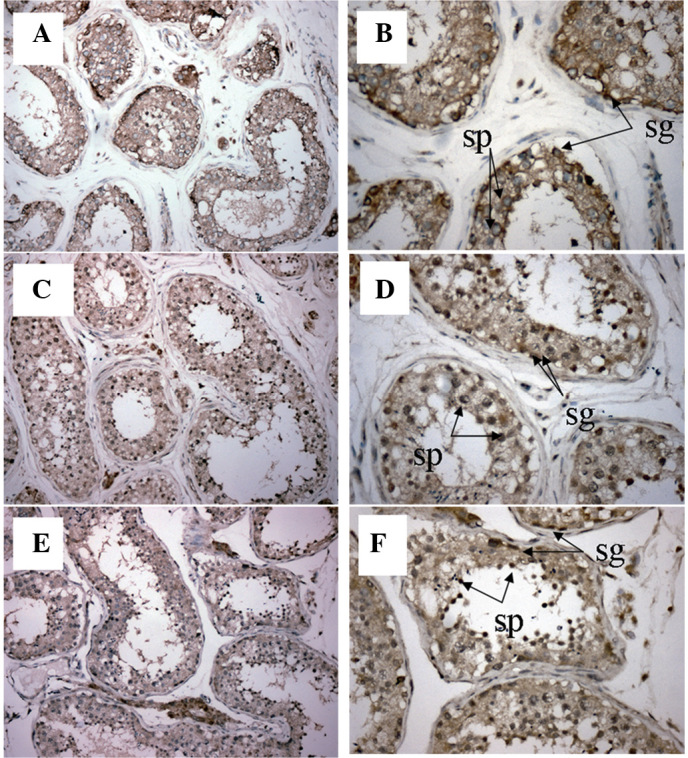
Immunohistochemical analysis of MAGE-A1, -A3 and -A11 expression in normal human testis tissue. Magnification: (A) MAGE-A1, ×200; (B) MAGE-A1, ×400; (C) MAGE-A3, ×200; (D) MAGE-A3, ×400; (E) MAGE-A11, ×200; and (F) MAGE-A11, ×400. In the normal testis tissue, MAGE-A1, -A3 and -A11 were mainly expressed in the primary spermatocytes (Sp) and spermatogonia (Sg). MAGE, melanoma-associated antigen.

**Figure 2.**
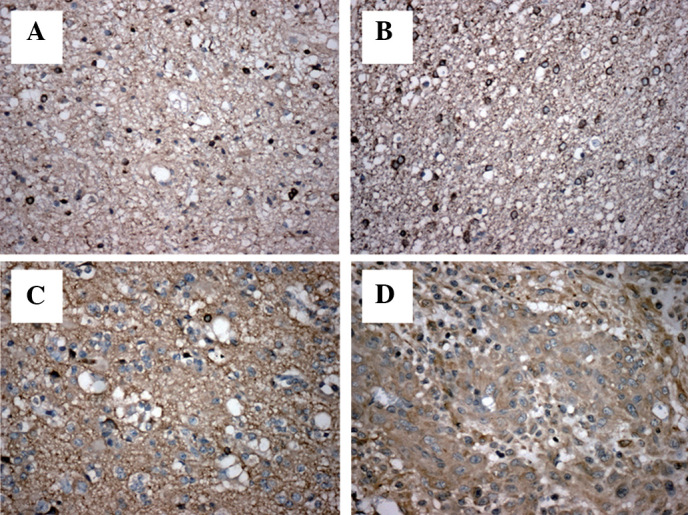
Immunohistochemical analysis of MAGE-A1 expression in the various pathological grades of glioma. (A) Grade I; (B) grade II; (C) grade III; and (D) grade IV. Magnification, ×400. MAGE, melanoma-associated antigen.

**Figure 3.**
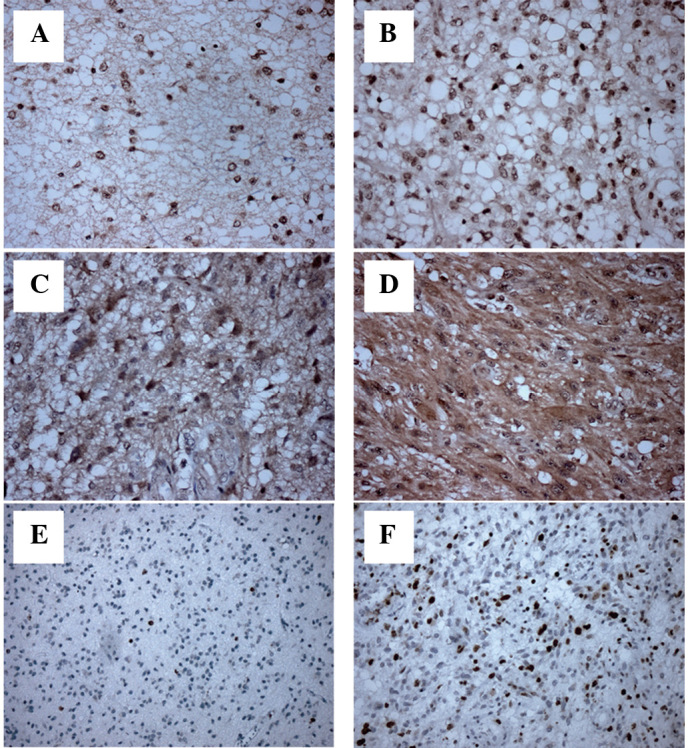
Immunohistochemical analysis of MAGE-A11 expression and ki-67 protein expression in the various pathological grades of glioma. (A) Grade I; (B) grade II; (C) grade III; (D) grade IV (magnification, ×400). (E) Low ki-67 expression; (F) high ki-67 expression (magnification, ×200). MAGE, melanoma-associated antigen.

In the normal human testis tissue, MAGE-A1 was mainly located in the cytoplasm and membrane. However, MAGE-A3 and -A11 were observed in the cytoplasm and nuclei of the primary spermatogonia and spermatocytes (Fig. 1). In the grade I–II gliomas, MAGE-A1 (Fig. 2A and B) was expressed mainly in the cytoplasm and membrane, while MAGE-A3 (data not shown) and -A11 (Fig. 3A and B) were expressed in the nuclei of the tumor cells. In the grade III–IV gliomas, MAGE-A1 (Fig. 2C and D) was mainly detected in the cytoplasm, while MAGE-A3 (data not shown) and -A11 (Fig. 3C and D) were mainly found in the cytoplasm and nuclei of the tumor cells.

The expression of MAGE-A1, -A3 and -A11 was not detected in the 15 normal brain tissues (data not shown). Out of the 78 glioma specimens, 50 (64.1%), 40 (51.3%) and 45 (57.7%) exhibited high expression levels with MAGE-A1, -A3 and -A11 antibodies, respectively (Table I). In addition, the frequencies of these antigens in the gliomas with various pathological grades were not accordant (Table II). The expression levels of MAGE-A1 (P= 0.000) and -A11 (P= 0.000) in the various pathological grades were significantly different and increased with the pathological grade. However, there was no significant difference between the MAGE-A3 expression level and the pathological grade.

### Associations between the expression levels of MAGE-A1, -A3 and -A11 and the clinicopathological characteristics of glioma patients

The association between the expression levels of MAGE-A1, -A3 and -A11 and the patients’ clinicopathological characteristics are shown in Table III. No associations were observed among age, gender, KPS score and the expression levels of MAGE-A1, -A3 and -A11. However, significant correlations were detected between the expression of MAGE-A1 (P=0.000) and MAGE-A11 (P=0.030) and the glioma pathological grade. There were no significant associations between MAGE-A3 (P=0.069) expression and tumor grade.

### Associations between MAGE-A1, -A3 and -A11 expression levels and the prognosis of glioma patients

Among the 78 glioma patients, the median survival time was 26 months and the one-, three- and five-year survival rates were 71.79, 37.16 and 21.56%, respectively.

**Figure 4.**
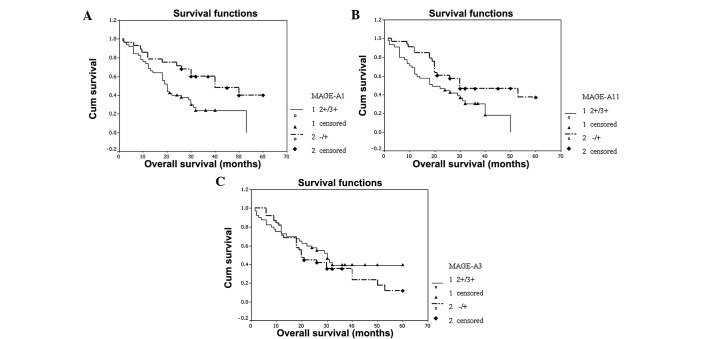
Kaplan-Meier curves showing overall survival (OS) with regard to (A) MAGE-A1, (B) MAGE-A11 and (C) MAGE-A3 protein expression levels. The patients with high MAGE-A1 and -A11 expression levels (2+/3+) exhibited significantly poorer outcomes compared with those with low expression levels (−/+), although no significant difference was detected between the two MAGE-A3 expression groups. MAGE, melanoma-associated antigen.

The OS of the patients in the MAGE-A1, -A3 and -A11 groups with high and low expression was examined and statistically significant differences were only observed between the MAGE-A1 (P= 0.005) and -A11 (P= 0.019) subgroups. The survival time of the patients with high expression levels of MAGE-A1 or -A11 was significantly lower compared with the patients with low expression levels (Fig. 4). In the univariate analysis, high pathological grade (P=0.000), low KPS score (P=0.000), decreased age (P=0.014), high ki-67 labeling index (P=0.050) and high MAGE-A1 (P=0.005) and -A11 (P=0.019) expression levels were correlated with poor patient outcomes (Table IV). Further assessment with Cox’s multivariable analysis showed that high pathological grade (P=0.000), low KPS score (P= 0.000) and high MAGE-A1 (P=0.007) and -A11 (P=0.010) expression levels were statistical predictors of poor OS (Table V). Additionally, the ki-67 labeling index was positively correlated with the expression of MAGE-A1 (P=0.026) and MAGE-A11 (P=0.008; Table VI).

## Discussion

CTAs are considered to be ideal targets for immunotherapy in tumors due to their characteristic expression pattern. The MAGE-A family, a subgroup of CTA, has been studied widely. MAGE-As are expressed in various types of cancer, including breast, lung, prostate and oral squamous cell carcinoma and bladder cancer (18–21). However, information concerning the expression of the MAGE-A family in glioma is limited and conflicting. Jacobs *et al* (22) analyzed cancer-germline gene expression, including that of MAGE-A1, -A2, -A3, -A4, -A6, -A10, -A12 and -C2, NY-ESO-1 and GAGE-1, 2 and 8 in 50 pediatric brain tumors (including 36 gliomas) using real-time PCR, and noted that 67% of astrocytic tumors expressed one or more cancer-germline genes. Another study (14) investigated the composite expression of CTA and MDA in malignant glioma using RT-PCR and showed that the frequencies of MAGE-A3, -A1 and -A4 expression were 22, 16 and 7%, respectively. Chi *et al* (23) observed that 38 and 33% of glioma tissues expressed MAGE-A1 and MAGE-A3, respectively, at the RNA level. Sahin *et al* (24) reported that only 8% of glioma specimens expressed MAGE-A3, while Saikali *et al* (25) demonstrated that the MAGE-A3 mRNA expression frequency in glioblastoma multiforme was 42%. Only a few studies have investigated MAGE-A antigens with immunohistochemistry or western-blot analysis. Our group previously investigated the expression of MAGE-A10 and -A11 in breast cancers and showed that they were tumor-specific antigens and that MAGE-A11 expression was a prognostic factor for a poor patient outcome (26). Although certain studies have investigated the expression of MAGE-A1 and -A3 in glioma, few have demonstrated the association between the expression levels and prognosis or cell proliferation status. There have been no studies on the expression of MAGE-A11 in glioma and its association with cell proliferation status or prognosis.

In the present study, the expression levels of the MAGE-A1, -A3 and -A11 proteins were evaluated with immunocytochemistry in glioma specimens, using normal testis tissues as positive controls and normal brain tissues as negative controls. It was revealed that MAGE-A1, -A3 and -A11 were expressed in the majority of gliomas. Furthermore, the 15 normal brain tissues showed no expression of MAGE-A1, -A3 and -A11, which was consistent with the CTA expression pattern. This suggested that MAGE-A1, -A3 and -A11 may potentially be used as ideal targets in immunotherapy for glioma (26–28).

Ki-67, a gene expressed during G1, S and G2 phases of the cell cycle (29–31), with a peak during mitosis an absence in the G0 phase, is often measured using MIB-1 antibody as a labeling index for evaluating the proliferation status of numerous types of cancer (32,33). The expression of the ki-67 protein has been correlated with disease aggressiveness and survival time in glioma patients (34–36). To investigate the association between the expression of MAGE-A1, -A3 and -A11 and the prognosis of patients with glioma, the expression of the ki-67 protein was also observed in the same glioma specimens in the present study. The results showed that the expression levels of MAGE-A1 and -A11 were positively correlated with the ki-67 labeling index, suggesting that MAGE-A1 and -A11 may be involved in tumor cell proliferation (37).

The survival analysis in the present study showed that the pathological grade, KPS score and expression levels of MAGE-A1 and -A11 were significantly correlated with patient outcome, while no association was observed between MAGE-A3 expression and prognosis. The survival of patients with high expression levels of MAGE-A1 and -A11 was significantly lower compared with patients with low expression levels, suggesting that MAGE-A1 and -A11 were potential factors for a poor prognosis for glioma. Certain studies have reported an association between MAGE-A1 and -A11 expression and tumor prognosis. Bodey *et al* (13) suggested that MAGE-A1 may be an indicator of childhood astrocytoma progression, although Grau E *et al* (38) showed that the MAGE-A1 expression was associated with a good prognosis in neuroblastoma. Our group previously detected the expression of MAGE-A11 in breast cancers and observed that it was a prognostic factor for poor patient outcome (26). There have been no previous reports concerning the prognostic significance of MAGE-A1 and -A11 in adult glioma. In the present study, high MAGE-A1 and -A11 expression levels in the patients with glioma were significantly correlated with poor OS and a high ki-67 labeling index. Consequently, we considered that MAGE-A1 and -A11 may be prognostic markers of poor outcome in glioma patients. Additionally, in the present results, increased pathological grades and low KPS scores were correlated with worse outcomes, which was consistent with previous studies (35,39).

In summary, the present preliminary study showed that MAGE-A1, -A3 and -A11 are expressed in glioma and that MAGE-A1 and -A11 expression levels increase with the pathological grade. The patients with high MAGE-A1 and -A11 expression levels had poor OS and high ki-67 labeling indices. The results suggest that MAGE-A1, -A3 and -A11 may be used as ideal targets in immunotherapy for glioma, and that MAGE-A1 and -A11 may be potential markers of a poor prognosis for glioma. However, the present study had a number of limitations. The number of patients was relatively small, which may have affected the statistical results. As it was a retrospective study, only expression at the protein level was detected. The prognostic role of MAGE-A1 and -A11 should be investigated in a greater number of glioma patients and using a greater number of methods. The mechanism responsible for MAGE-A1 and -A11 expression in tumorigenesis and biological functions requires further evaluation.

## Figures and Tables

**Table I.** Expression levels of MAGE-A1, -A3 and -A11 in glioma and normal brain tissues.

Type of tissue	MAGE-A1		χ^2^	P-value	MAGE-A3		χ^2^	P-value	MAGE-A11		χ^2^	P-value
	−/+	2+/3+			−/+	2+/3+			−/+	2+/3+		
Glioma	28	50	20.796	0.000	38	40	13.498	0.000	33	45	16.767	0.000
Normal brain tissue	15	0			15	0			15	0		

MAGE, melanoma-associated antigen.

**Table II.** Expression levels of MAGE-A1, -A3 and -A11 in various pathological grades of glioma.

Grade	n	MAGE -A1				χ^2^	P-value	MAGE -A3				χ^2^	P-value	MAGE -A11				χ^2^	P-value
		−	+	2+	3+			−	+	2+	3+			−	+	2+	3+		
I	9	6	2	1	0	^*^	0.000	2	2	3	2	3.144	0.958	6	2	0	1	30.321	0.000
II	16	2	9	5	0			5	4	4	3			2	5	4	5		
III	17	2	2	4	9			5	5	5	2			4	6	2	5		
IV	36	1	4	12	19			6	9	12	9			0	8	8	20		

* Fisher’s exact test. MAGE, melanoma-associated antigen.

**Table III.** Associations between MAGE-A1, -A3 and -A11 expression levels and the clinicopathological characteristics of patients with glioma.

Clinicopathological parameters	MAGE-A1		χ^2^	P-value	MAGE-A3		χ^2^	P-value	MAGE-A11		χ^2^	P-value
	−/+	2+/3+			−/+	2+/3+			−/+	2+/3+		
Age (years)												
≥50	16	21	1.651	0.199	19	18	0.195	0.658	17	20	0.382	0.537
<50	12	29			19	22			16	25		
Gender												
Male	17	28	0.163	0.686	20	25	0.778	0.378	19	26	0.000	0.986
Female	11	22			18	15			14	19		
Pathological grade												
Glioma I–II	19	6	25.714	0.000	13	12	0.159	0.690	15	10	4.718	0.030
Glioma III–IV	9	44			25	28			18	35		
KPS score												
≥80	8	13	0.060	0.806	3	18	13.637	0.000	8	13	0.209	0.648
<80	20	37			35	22			25	32		

MAGE, melanoma-associated antigen.

**Table IV.** Univariate analyses to evaluate the effect of variables on median survival and survival rate for 78 patients with glioma.

Variable	Cases	Median survival (months)	Survival rate (years)			χ^2^	P-value
			1	3	5		
Age (years)							
≥50	37	40	83.78	50.61	25.30	6.050	0.014
<50	41	19	60.98	22.50	22.50		
Gender							
Male	45	30	75.56	40.73	27.15	1.181	0.278
Female	33	20	66.67	32.49	14.44		
Pathological grade							
Glioma I–II	25	50	100.00	76.55	47.63	24.090	0.000
Glioma III–IV	53	18	58.49	18.57	9.29		
KPS score							
≥80	21	-	85.71	69.84	69.84	12.610	0.000
<80	57	20	66.67	25.20	9.45		
MAGE-A1 expression							
−/+	28	40	78.57	59.87	39.92	7.960	0.005
2+/3+	50	20	68.00	23.69	0.00		
MAGE-A3 expression							
−/+	38	20	71.05	35.49	11.83	1.060	0.304
2+/3+	40	30	72.50	39.20	39.20		
MAGE-A11 expression							
−/+	33	30	84.85	46.65	37.32	5.550	0.019
2+/3+	45	19	62.22	30.41	0.00		
Ki-67 expression							
<10%	17	50	88.24	59.13	0.00	3.770	0.050
≥10%	61	20	67.21	31.18	23.39		

MAGE, melanoma-associated antigen; KPS, Karnofsky performance scale.

**Table V.** Multivariate Cox’s regression analysis for patients with glioma.

Factor	Variable	B	SE	Wald	P-value	OR	Low	Upper
MAGE-A1 expression	−/+ or 2+−3+	0.875	0.325	7.248	0.007	2.399	1.269	4.538
Pathological grade	I–II or III–IV	1.728	0.398	18.881	0.000	5.630	2.582	12.276
MAGE-A11 expression	−/+ or 2+−3+	0.800	0.311	6.618	0.010	2.225	1.210	4.092
KPS score	<80 or ≥80	−1.678	0.448	14.008	0.000	0.187	0.078	0.450

MAGE, melanoma-associated antigen; KPS, Karnofsky performance scale; B, regression coefficient; SE, standard error; OD, odds ratio; Low, lower limit; Upper, upper limit.

**Table VI.** Associations between expression levels of MAGE-A1, -A3 and -A11 and ki-67 labeling index.

Ki-67 labeling index	MAGE-A1		χ^2^	P-value	MAGE-A3		χ^2^	P-value	MAGE-A11		χ^2^	P-value
	2+/3+	±			2+/3+	±			2+/3+	±		
<10%	7	10	4.965	0.026	6	11	2.224	0.136	5	12	7.123	0.008
≥10%	43	18			34	27			40	21		

MAGE, melanoma-associated antigen.
